# Neural signatures of online and offline motor learning: An ALE meta-analysis

**DOI:** 10.1162/imag_a_00457

**Published:** 2025-01-24

**Authors:** Gabriel Byczynski, Elva Arulchelvan, Yvette Grootjans, Iulia-Mara Scarlat, Simone Brady, Sophie Kamdar, Sven Vanneste

**Affiliations:** School of Psychology, Trinity College Dublin, Dublin, Ireland; Lab for Clinical & Integrative Neuroscience, Trinity Institute for Neuroscience, School of Psychology, Trinity College Dublin, Dublin, Ireland; Global Brain Health Institute & Institute of Neuroscience, Trinity College Dublin, Dublin, Ireland

**Keywords:** motor learning, meta-analysis, fMRI, motor task, motor sequence, visuomotor learning, sequence learning, offline learning, online learning

## Abstract

Neural activation patterns underlying motor learning that are captured using functional imaging can only reflect the patterns occurring at a given moment. Motor learning is known to comprise many processes which are variably biologically or temporally distinct. In order to improve the understanding of how regional activation patterns may vary across different mechanisms of motor learning, we performed an ALE meta-analysis of imaging studies that directly compares online and offline motor learning. Using coordinate-based meta-analysis methods and independent review, 1777 studies were returned from 3 databases. Thirty-eight studies investigating motor task learning met the inclusion criteria, were allocated as either online or offline learning based on their scanning placement, and revealed both unique and overlapping regional activation/deactivation patterns. We identify activation changes in regions that are consistent for online learning and offline learning. Our findings concur with those of previous meta-analyses investigating online motor learning, and find support for previous theories surrounding the networks involved in consolidation and offline processes in motor learning. Shared activation between online and offline motor learning was found in the supplemental motor area and somatosensory cortex, highlighting regions which are continually involved in both processes, and identifying those which may be differentially modulated to alter motor learning outcomes.

## 1 Introduction

Brain imaging research has been used to uncover the neurobiological underpinnings of numerous cognitive functions and processes. When performed alongside a task, imaging allows researchers to visualize the activity of the brain, correlating activity with function and process. During motor tasks, this method uncovers the activation changes and processes that underly the domains of movement, working memory, error detection, adaptative learning, and attention (Seidler et al., 2012;Shadmehr et al., 2010).

Specific to motor learning, there are both continuous and transient neural processes which govern how the brain learns movements (Verhoeven & Newell, 2018). Although there is variability in the naming of the processes, there is consensus that motor learning begins with acquisition (sometimes called learning or motor adaption), which is followed by consolidation, and then a longer and less defined stage in which the brain retains the learned skill, sometimes referred to as retention (Luft & Buitrago, 2005). At the behavioural level, acquisition is the initial learning of the task, either during (fast) or between sessions (slow) (Luft & Buitrago, 2005), or alternatively described as occurring over three phases: the cognitive, the associative, and the autonomous (Marinelli et al., 2017). Functionally, motor learning acquisition has been correlated with activation of the primary motor regions, sensory regions, cerebellum, basal ganglia, prefrontal cortices, cingulate cortices, and parietal cortices, dependent upon the type of task being used (Ghilardi et al., 2000). In the domain of adaptive motor learning, acquisition manifests as the combined influence of an internal forward model, continued adaptation to motor targets, and the integration of information from commands and its subsequent sensory feedback (Shadmehr et al., 2010). Such a system relies on the same regions which have previously been associated with motor learning. Specific systems including the cortico-striatal and cortico-cerebellar are implicated as being essential for acquisition and performance of learned motor skills (Doyon & Benali, 2005), albeit there is no meta-analysis to our knowledge that has assessed the consistency of activation of the cortico-striatal network across multiple forms of motor learning experiments, as it pertains to the adaptative motor learning framework.

In*consolidation*, the stage which follows the initial learning of the task, the learned skill is consolidated (however is susceptible to interference). It has been illustrated that the M1 region is involved in motor consolidation, with one study showing that brain activity shifts from prefrontal to premotor, parietal, and cerebellar regions (Muellbacher et al., 2002). Research has implicated other consolidation-related structures including the striatum, somatosensory cortex, lobule VI of the cerebellum, and the caudate nucleus (Debas et al., 2010;Doyon & Benali, 2005;Wali, 2020). Following acquisition via adaptive processes, the newly acquired internal model becomes increasingly consolidated and thus resistant to interference by a competing model (Shadmehr & Brashers-Krug, 1997).

Accordingly, motor learning acquisition (via the processes of adaptation and development of the internal model) is a form of online learning, while learning effects during motor consolidation would be considered offline learning (whereby the internal model becomes increasingly resistant to retrograde interference). It has been shown that these forms of learning are governed in some ways by similar regions (Ehsani et al., 2016), and in other instances, different ones (Lacroix et al., 2019;Pollok et al., 2020). A pattern then emerges where regional activation changes across motor learning processes, albeit not necessarily for the same reasons. One consistent network which appears engaged in both online and offline motor learning is the cerebral network, which includes the M1, pre-motor cortex, supplementary motor area (SMA), basal ganglia, prefrontal cortex, posterior parietal cortex, and cerebellar regions (SeeFig. 1inDahms et al. (2020)for a clear schematic of how this network is engaged over motor learning stages).

**Fig. 1.**
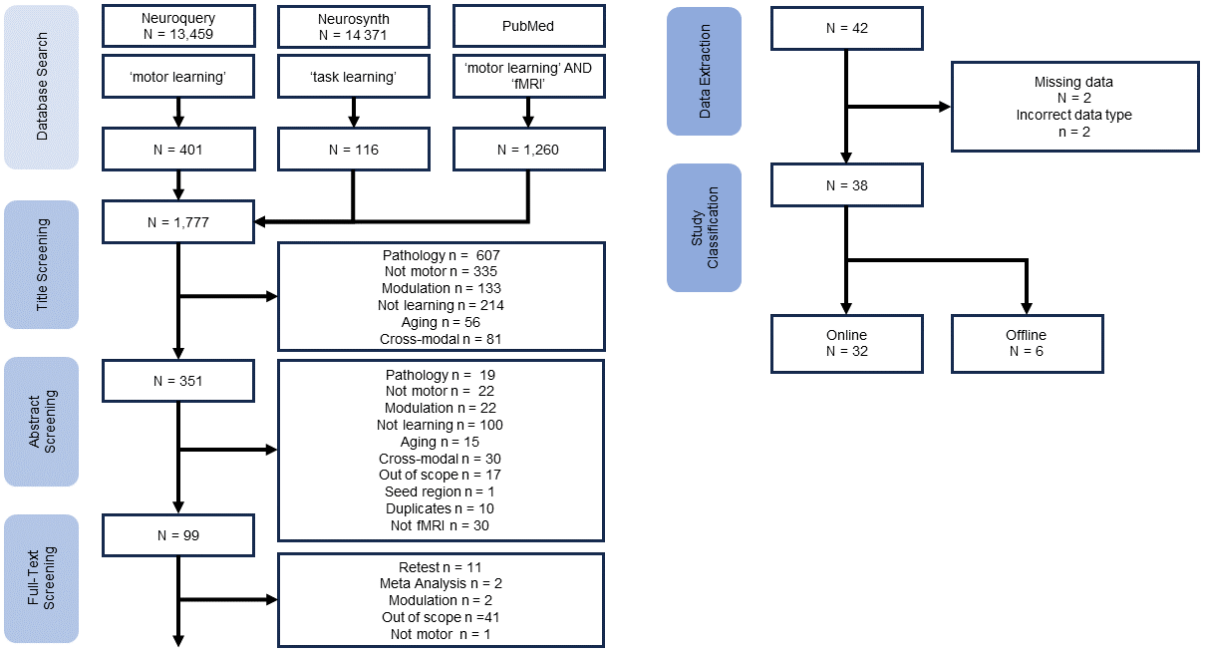
Pipeline of study inclusion from database search, title screening, abstract screening, full-text screening, and categorization. Values for exclusions and inclusions are separated by database searched. Initial database search was automated, all screening and categorization were manual.

Although there is extensive work in motor learning, with a strong understanding of the brain activation patterns that occur during different timepoints in the learning process, there are few studies which broadly discuss the neural correlates of online and offline learning, as they relate to motor learning, and the current theories surrounding their mechanisms. One known review (Di Rienzo et al., 2016) investigated online and offline performance gains during motor imagery, as it relates to the promotion of motor learning. They conclude that online and offline learning contribute to performance, highlighting that the neurophysiological correlates of offline motor imagery practice are generally unexplored. We, therefore, feel it important to produce a similar analysis which investigates online and offline motor learning correlates across previous motor learning studies, in order to validate and judge the consistency of activation across a heterogeneous literature of task choices, demographics, and scanning points. This meta-analysis would then seek to relate the neural signatures to current frameworks in motor learning in order to not only support regional activation, but also relate online and offline differences in the context of current motor learning theory (Gupta & Rickard, 2024;Shadmehr et al., 2010).

We believe that it is valuable to create a generalized picture of a theoretical motor learning experiment where the online and offline correlates of motor learning can be effectively compared and contrasted using meta-analytical methods. By performing a coordinate-based meta-analysis (CBMA) on studies that looked at different periods of motor learning, we can create a binary representation of motor learning across online and offline stages that may better capture the transient or persisting nature of specific activation patterns and processes. Functional-imaging meta-analyses are becoming increasingly popular, additionally with the support of large-scale neuroimaging databases such as NeuroSynth (Yarkoni et al., 2011) and NeuroQuery (Dockès et al., 2020). Indeed, meta-analytical environments such as NiMARE (Salo et al., 2023) further organize and simplify the meta-analytical process, by combining numerous tools and databases into a single library and allowing users to access a larger scale of resources to complete rigorous and reproducible analyses. The objective of this study is, therefore, to use a coordinate-based meta-analysis with activated-likelihood estimate (CBMA ALE) approach to investigate motor learning studies available on the NeuroQuery, NeuroSynth, and PubMed databases.

In the following meta-analysis, we aim to produce an estimated representation of how brain activation patterns during two aspects of motor learning (online and offline) may transition or persist, which will provide context and clarity to how regional activation/deactivation may occur during motor learning. We will follow an approach that categorizes studies based on this criterion, and investigates unique and overlapping patterns between online and offline learning. This work intends to address the manifestation of neural signatures representing online and offline learning, allowing for a clear comparison between the regions implicated, across a variety of motor learning tasks and paradigms. Identifying consistent regional activation will also test current hypothesis surrounding the circuitry involved in acquisition and consolidation, and place regional activation signatures in the context of online and offline motor learning theories, and their synthesis with adaptive motor control (Gupta & Rickard, 2024;Shadmehr et al., 2010). Accordingly, our results aim to be a generalizable representation of motor learning correlates during two critical periods in the process. We also aim to give context to the growing number of studies which investigate motor learning, and even stimulation applications (e.g., tDCS, tACS, TMS) to modulate motor learning, in order to provide insight into regional specificity in both online and offline targets for modulation, regardless of the task.

## 2 Methods

This meta-analysis sought to estimate activation changes across two motor learning processes, by taking existing motor learning stages, and comparing and contrasting activation patterns between the scanning points. We chose to subdivide motor learning into either online or offline timepoints (either scanning during the task, or after the fact). Accordingly, we use the scanning timepoint as the categorizing factor. Scanning concurrent with the task learning would capture signatures of online learning (e.g., acquisition) or reflect neural processes of motor task performance, and scanning after the task or during rest would capture offline learning or the after-effects of the learning (e.g., consolidation or retention). We also opted not to reduce the study by criteria by the task under investigation, in order to (1) generalize our findings to motor learning processes non-specific to a given task and (2) to allow more diverse study inclusion. No ethics approval was required for this study since no primary data were collected.

### 2.1 Study Inclusion

The pipeline of study inclusion criteria is illustrated inFigure 1. All studies included had publication year, authors, title, and reported activation/deactivation peaks and coordinates listed. Studies were only accepted if they could be found online (e.g., internet search, database search). All studies included were in English.

Studies included in the meta-analysis were required to have a clear study design, and define when the imaging took place in the course of the experiment. This was necessary to confirm what the reported peaks reflected (e.g., during or after task). Pathology groups or medication groups were excluded to ensure that coordinates reported were the result of motor task learning, and not the result of impaired or altered motor learning. In addition, studies with age extremes (i.e., less than 18 years) or studies that explicitly investigated “aging effect” were not included, as activation changes due to ageing or maturation may not represent motor learning in a comparable way (Zapparoli et al., 2022). Studies must also have a clear motor learning component (i.e., studies purposefully investigating motor tasks). Studies that included motor tasks without any learning aspect were excluded, since peaks reported in these studies may not reflect a learning effect. Studies included were required to be research articles, so reviews and meta-analyses were not included. Studies that were beyond the scope of the meta-analysis (e.g., non-human, ROI, investigations of subject variability) were excluded in order to refine the analysis to group-level activity reflecting motor learning. During extraction, studies were excluded if data were missing (i.e., peak value, coordinate values, cluster size), or if the data were of an incompatible type for a coordinate-based meta analysis (i.e., T-score instead of Z-score).

### 2.2 Database and search

The databases, NeuroSynth (https://neurosynth.org/) (Yarkoni et al., 2011) and NeuroQuery (https://NeuroQuery.org/) (Dockès et al., 2020) were searched through the NiMARE python package (NiMARE v0.0.11 (RRID:SCR_017398 (Salo et al., 2023)). PubMed was used to supplement studies which may not have been indexed by either database, in order to a more robust search methodology which is not restricted by the imaging databases.

In NeuroQuery, the abstract search term “motor learning” was used. Following the initial search, to widen the search results using a varied search term, the NeuroSynth database was queried using the term “motor task”. The search terms differ because, while this meta-analysis intends to focus on motor learning, NeuroSynth did not support motor learning as a search term. It is expected that its use as a search term will result in a larger number of rejections for the dataset returned, since the search term was less specific to motor learning. The search term was limited to English, since to our knowledge the NeuroQuery and NeuroSynth databases only contain studies written in English. For more information about the NeuroQuery database, and its comparison with NeuroSynth as a meta-analytical search software, see the paper by Dockès and colleagues (Dockès et al., 2020). PubMed was, therefore, used to search studies which may not have been indexed within either NeuroQuery or NeuroSynth, but which may meet criteria.

All databases were searched in March 2024. The initial database search was automated, and following the initial query, a manual selection process was carried out in two stages: (1) To determine inclusion or exclusion of each study and (2) to categorize the included studies. At both stages, three independent reviewers screened each study for the inclusion/exclusion criteria. The title, abstract, and then full text of each paper were subsequently used to verify no clear exclusion factors, and study inclusion was then verified with the body of the text. If both reviewers agreed on a decision to include the study, it was passed to the second stage of categorization. Disputes were moderated by a third party. If all reviewers agreed that the study did not meet inclusion criteria, or had an exclusion criterion, then the study was rejected. In stage 2, the same process was carried out, where two independent reviewers use the categorization criteria to determine which category the study was to be included. If there was disagreement in decision, the study was given to the third independent reviewer, who moderated the decision on categorization.

### 2.3 Study data collection

Two reviewers independently gathered data from each report, and disagreements were handled with a third independent reviewer. All data were validated by a third reviewer. The number of peaks and their coordinates from each study were automatedly derived using the NiMARE package, and all other study information was sourced from the literature. Due to the nature of this meta-analysis, the peaks and their coordinates are designated as the main outcome variable from the studies analyzed.

Data included the year of publication, the sample size, and type of task used, were reported, along with mean age, age range, and right handedness, and number of females in each sample (as available). When studies did not report a value, it was left blank and not inferred. In the instance where a study stated the initial sample demographic, but then reported withdrawals/data exclusions without updated demographic information, the corresponding demographic information was not inferred, and instead was left blank.

### 2.4 Synthesis and analysis methods

An activation likelihood estimate algorithm was used to perform a CBMA on the studies. Briefly, an ALE analysis models the activation based on coordinates and peak values, for each study individually. The created activation maps are combined to a single map based on a voxel-based assignment of each ALE value, and compared with a null distribution, returning a z-map of the activation for each group/subgroup. It is important here to note that the data presented here do not strictly represent activation, but rather the coordinates and peaks reported are simply values at which there was significant increase or decrease in activation. Thus, while the ALE describes, in name, “activation,” the results represent the implication of regions in general—rather than*increased*activation. In an ALE analysis, a Gaussian kernel is used to account for the spatial uncertainty of the peaks by smoothing the values across neighbouring voxels. The width of the kernel is proportional to the sample size of the study, accounting for the likelihood that studies with larger sample sizes are a better indicator of the true location of a peak (Eickhoff et al., 2009). The ALE analysis was performed on each group (i.e., online and offline), with an uncorrected significance threshold of*p*< 0.001, following previous ALE papers (Grosbras et al., 2012;Hétu et al., 2013). Cluster volume for both ALE analyses was set to a minimum of 100 mm^3^, although for completeness, visualizations include all clusters regardless of cluster size. Those clusters which met the minimum size were subsequently mentioned and discussed.

The resulting ALE statistical maps were used in a conjunction analysis, in which each online and offline ALE maps were compared to identify regions that have significant overlapping activation/deactivation. Regions were identified with the Automatic Anatomical Labeling (AAL) template (Tzourio-Mazoyer et al., 2002) and MNI coordinates. Lasty, in order to investigate bias in activation patterns shown, a jackknife analysis was performed, whereby studies are systematically removed to determine how each cluster is impacted. This allows for an identification of clusters which are consistently activated across studies opposed to clusters which are dependent on a smaller subset of studies.

## 3 Results

### 3.1 Database search

The results of the NeuroQuery, NeuroSynth, and PubMed databases searches yielded 401, 116, and 1260 studies, respectively (1777 total). Overall, 38 studies were retained. Of the accepted studies, 32 were categorized as online learning and 6 as offline learning (Table 1).

**Table 1.** Studies included in the meta-analysis, with metadata and categorization.

Type	Study	NSS	NF	NRH	Age range	Mean age	Task used
Online	Heun et al. (2004)	10	5	10	23-25	23.7	SRTT
	Oishi et al. (2005)	18	11	18	21-39	-	SRTT
	Penhune and Doyon (2005)	12	6	12	-	24.8	TMST
	Olson et al. (2006)	10	5	10	19-40	25	SRTT
	Macintosh et al. (2007)	12	7	12	-	29	Finger movement
	Anguera et al. (2007)	11	5	11	18-30	21.4	Spatial task
	Grafton et al. (2008)	10	-	-	-	-	Tracking task
	Tamás Kincses et al. (2008)	15	7	15	25-32	27.5	SRTT
	Rémy et al. (2008)	12	6	12	-	23.6	Coordination task
	Albouy et al. (2008)	90	-	90	19-28	-	SRTT
	Schlund et al. (2008)	10	-	10	18-50	-	Operant conditioning task
	Fernández-Seara et al. (2009)	14	7	14	-	31	SRTT
	Orban et al. (2010)	32	17	32	-	23.9	SRTT
	Tomassini et al. (2011)	12	9	12	-	30.1	Tracking task
	Orban et al. (2011)	12	6	12	-	26.5	SRTT
	Pammi et al. (2012)	17	-	17	-	-	SRTT
	Watanabe et al. (2011)	15	0	15	19-47	22.8	Imitation task
	Lefebvre et al. (2012)	20	11	20	18-62	33.9	Cursor task
	Melcher et al. (2013)	33	18	33	-	24.7	Go/NoGO
	Lissek et al. (2013)	18	11	18	23-34	25.66	SRTT
	Cunningham et al. (2013)	24	20	24	-	25	Tracking task
	Tzvi et al. (2014)	17	10	-	20-32	-	SRTT
	Kornysheva and Diedrichsen (2014)	32	16	32	19-36	24.8	Motor sequence task
	Karim et al. (2017)	13	6	13	-	23.8	Motor sequence task
	Tracy et al. (2001)	5	3	5	21-27	23.6	Single finger opposition
	Drobyshevsky et al. (2006)	31	15	31	22-76	41	SRTT
	Sacco et al. (2009)	8	4	8	20.8-34.8	27	Finger movement
	Gheysen et al. (2010)	22	17	22	19-25	-	Serial colour matching task
	Arima et al. (2011)	13	0	13	24-35	27.3	Tongue-training task
	Heitger et al. (2012)	34	19	34	20-30	23	Bimanual coordination training
	Müller et al. (2002)	7	0	7	23-46	-	SRTT
	Bo et al. (2011)	14	8	14	-	21.4	SRTT
Offline	Gregory et al. (2014)	12	8	12	-	25	SRTT
	Vahdat et al. (2011)	13	-	13	21-44	-	Reaching task
	Gryga et al. (2012)	15	5	15	22-32	-	Sequential pinch force task
	Tung et al. (2013)	24	15	24	-	30	SRTT
	Sidarta et al. (2016)	22	14	22	-	22.5	Reaching task
	Sami and Miall (2013)	24	-	24	-	23.6	SRTT

SS = sample size, F = number of females, RH = number of right-handed participants, SRTT = serial reaction time task (or alternative), TMST = temporal motor sequencing task.

### 3.2 ALE results

**Fig. 2.**
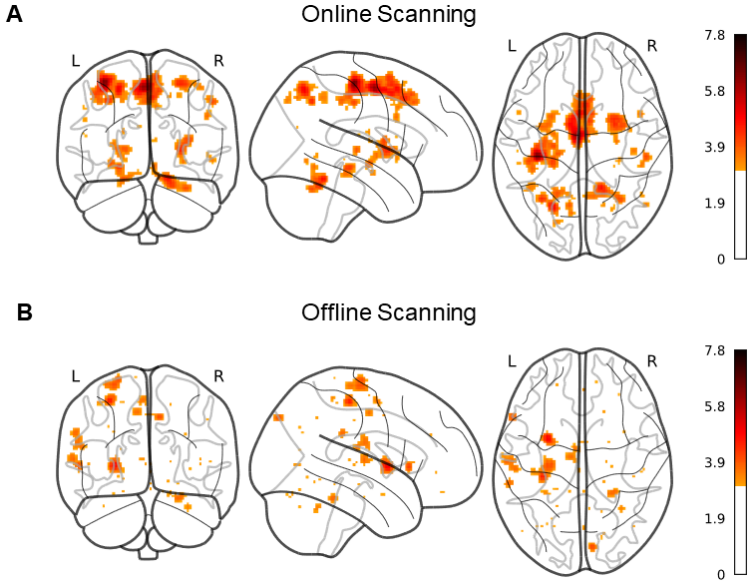
ALE results showing all significant clusters during (A) online and (B) offline motor learning. Colour bar represents magnitude of Z-statistic, and colouring threshold corresponds to*p*< 0.001.

The ALE returned 20 significant (*p*< 0.001) clusters greater than 100 mm^3^in volume in the online dataset and 11 in the offline subgroup. Significant clusters, regardless of size, are shown for online motor learning (Fig. 2A) and offline motor learning (Fig. 2B). Cluster MNI coordinates, peak statistic, size, and associated region are presented inTables 2and3. The ALE analysis of online motor learning studies revealed significant activations of frontal, parietal, and temporal gyri, the cerebellum, putamen, pallidum, and thalamus, among other regions. The offline motor learning analysis revealed ALE clusters in the regions of the cerebellum, putamen, cingulum, pre- and post-central gyri, frontal gyri, and supramarginal gyri.

**Table 2.** Significant clusters from ALE analysis of online motor learning.

Cluster ID	X	Y	Z	Peak statistic	Cluster size (mm ^3^ )	Region (AAL)
1	−4	−8	54	6.177475	7960	L Supplementary motor area
2	−36	−24	58	6.420258	4216	L Precentral gyrus
3	16	−50	−20	4.754861	2008	L Cerebellum, lobule 4/5
4	−24	−64	52	5.034228	1544	L Superior parietal gyrus
5	26	2	2	4.502371	1528	R Putamen
6	26	6	56	4.476016	1400	R Superior frontal gyrus
7	−24	−28	−12	4.224487	680	L Parahippocampal gyrus
8	−32	0	52	3.820151	584	L Middle frontal gyrus
9	−20	−54	−20	3.925206	536	L Cerebellum lobule 6
10	52	−24	42	4.040562	440	L Postcentral gyrus
11	−24	8	4	3.7921	408	L Putamen
12	−42	−50	46	3.475601	320	L Inferior parietal gyrus gyrus
13	−18	−22	4	3.875925	264	L Thalamus
14	−30	−12	−8	3.958692	248	L Putamen *
15	−10	−64	56	3.813831	216	L Precuneus
16	42	−20	54	3.511122	160	R Postcentral gyrus
17	-20	-78	44	3.437217	152	L Superior occipital gyrus
18	-22	-6	-2	3.602561	144	L Pallidum
19	48	10	30	3.596088	144	R Inferior frontal operculum
20	-28	-4	8	3.491422	136	L Putamen
21	-8	-70	46	3.597424	104	L Precuneus

* Region closest to coordinates.

AAL = Automated Anatomical Labelling Atlas 3.

**Table 3.** Significant clusters from ALE analysis of offline motor learning.

Cluster ID	X	Y	Z	Peak statistic	Cluster size (mm ^3^ )	Region (AAL)
1	−28	−20	68	4.004511	1128	L Precentral gyrus
2	−28	2	2	4.944095	912	L Putamen
3	−32	−28	54	4.537863	592	L Postcentral gyrus
4	−54	−22	18	3.648504	512	L Supramarginal gyrus
5	8	−84	42	3.961662	288	R Cuneus
6	−60	−16	8	3.729101	264	L Superior temporal gyrus
7	24	−42	−24	3.703366	256	R Cerebellum
8	−8	−12	46	3.609021	232	L Middle cingulum
9	−56	20	2	4.152583	224	Triangular part of the inferior frontal gyrus
10	−58	−34	30	3.702617	216	L Supramarginal gyrus
11	−4	−6	56	3.588852	184	L Supplemental motor area

AAL = Automated Anatomical Labelling Atlas 3.

### 3.3 Conjunction analysis

The results of the conjunction analysis revealed two clusters larger than 100 mm^3^that had significant shared activation changes. The clusters are shown inFigure 3, with the coordinates, peak statistic, and cluster size. Cluster 1 corresponded to the left postcentral gyrus, and cluster 2 corresponded to L supplemental motor area.

**Fig. 3.**
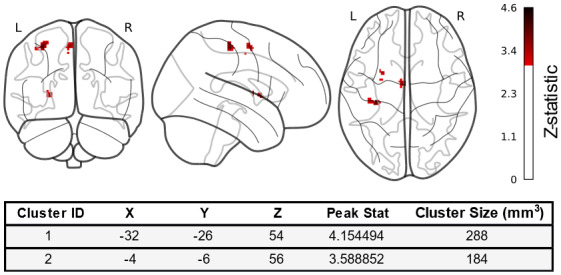
ALE results showing all significant shared clusters during both online and offline motor learning. Colour bar represents magnitude of Z-statistic (Peak Stat), and colouring threshold corresponds to*p*< 0.001. Peak State corresponding to Z-statistic is numerically represented with Cluster ID, MNI coordinates, and Cluster Size.

### 3.4 Evaluation of robustness

The results of the jackknife analysis showed that in each of the online and offline study groups, there was one cluster in which all studies included contributed activation. For the online group, a cluster with centre of mass at MNI coordinates [0, -20, 17] had contributions from all studies, corresponding to the midpoint between thalami. The offline group produced a cluster with centre of mass at MNI coordinates [-1, -23, 12] had contributions from all offline studies, likewise attributed to the midpoint between thalami. All cluster values are presented in theSupplemental Tables. Thus, our results illustrate that both online and offline scanning studies implicated activation changes in (bilateral) thalami.

## 4 Discussion

The analysis found that both online and offline learning studies had consistent activations of several regions encompassing known circuits in motor acquisition and consolidation, corroborating current regional implication in both motor adaptation and its subsequent consolidation. Shared activation between stages was also confirmed in the motor and somatosensory cortices. This finding represents novel evidence of consistent motor networks activated across a variety of motor learning paradigms, and which are specific to either online and offline motor learning. Our findings of offline motor learning correlates also provide empirical support for the cortico-striatal network’s involvement in motor learning, irrespective of the task (i.e., reaching, pinch force, SRTT), and the cerebellar–thalamic–cortical activation described during motor adaptive processes (Porrill & Dean, 2007).

### 4.1 Online motor learning

Findings from the analysis of studies that performed concurrent scanning and motor learning revealed that across both traditional serial reaction time task (SRTT) and visuomotor tasks, there was significant activation of previously described core regions associated with motor learning. Namely, in comparison with a recent quantitative meta-analysis of motor learning (Hardwick et al., 2013), we corroborate here that areas including the cerebellum, putamen, thalamus, precentral (M1), and parietal (SPL) regions are activated consistently during online motor learning experiments.

Placed in context of regional connectivity and motor learning circuitry, the regions implicated in our analyses are also supported by regions involved in motor system control, whereby M1 regions extend descending pathways to cerebellar regions and the basal ganglia, which in turn terminate on thalamic nuclei, creating a loop with output back to the M1 (Papale & Hooks, 2018). In the context of more recent framework, including that of adaptive motor control and learning, cerebellar involvement during online scanning would be consistent with the forward model of adaption, whereby consequences of motor commands are predicted and thus movement is corrected (Shadmehr et al., 2010). Accordingly, this movement could then be corrected via the thalamus, which is a region also consistently activated online here, projecting to the cortices (i.e., motor) (Shadmehr & Krakauer, 2008). Furthermore, we note extensive activation of the subcomponents of the basal ganglia including putamen and globus pallidus, which too have been proposed to play a role in correction during controlled movement in the motor cortex (Magdoom et al., 2011). It would then appear that across the numerous task parameters and designs included here, consistent activation of regions involved in the adaptative pathway framework (e.g., cerebellum, thalamus, cortex, putamen) supports its role in motor learning, and identifies further numerous cortical regions likely involved in further processing, and which have previously been shown necessary for correct motor memory (Ebrahimi & Ostry, 2024).

It is noted that much of the activation presented in our findings appears left-lateralized, however, we cannot draw conclusions about lateralization in motor learning, since we confirmed that 30 of the studies of online learning (93%) recruited participants who all boasted right-handed tendency (the remaining 2 studies did not clearly report handedness, and thus conclusions could not be drawn). Thus, it is likely that the pattern of lateralized activation is related to handedness (Andersen & Siebner, 2018).

### 4.2 Offline motor learning

The offline scanning analysis revealed that while regions such as the cerebellum, precentral gyrus, and putamen are activated in both the online and offline studies, there are several regions which were uniquely activated in the offline studies alone. The postcentral gyrus, supramarginal gyrus, cuneus, superior temporal gyrus, middle cingulum, inferior frontal gyrus, and supplemental motor area had clusters that were significantly activated during offline scanning. Interestingly, it has been shown in other work that regions such as the supramarginal gyri and postcentral gyri are indeed sensitive to training duration, and show activation changes during resting-state scans following motor learning (Ma et al., 2011). The cuneus has also been implicated during resting-state scans following motor learning, and has been suggested to be a part of a functional relationship with the sensorimotor areas (Roshchupkina et al., 2022), with additional evidence showing that resting-state cuneus functional activity correlates with motor learning (Mary et al., 2017). Pertaining to the offline regions identified here, the middle cingulum, putamen, post-central, pre-central, and cuneus have all been implicated as being a part of the cortico-striatal network (Debas et al., 2014). This network has been presented as a potential mechanism by which motor sequence consolidation occurs in the brain, as it has been shown that increased functional connectivity within the network relates to greater durability of motor trace through offline consolidation processes (Debas et al., 2014). We, therefore, find that many of the regions which are activated during resting-state and post-motor learning scans were identified as regions of a network related to consolidatory processes in motor learning. Similarly to online motor learning, consolidation of motor memory in the context of adaptive motor learning framework would also explain cerebellar activation when arguably there is no movement to be corrected, and this dually implicates the consolidatory processes which may similarly be occurring during post-learning offline scanning. Animal studies (Attwell et al., 2002) have shown that the cerebellum is equally involved in retention, positing that adapted behaviour immediately following acquisition is dependent on cerebellar plasticity (Krakauer & Shadmehr, 2006).

It is important to note that offline scanning timepoints may not capture strictly “offline motor learning” processes as with online motor learning. This is due to the fact that some tasks do not show offline improvements, such as the explicit SRTT (Tunovic et al., 2014). Despite this, we highlight that reported peaks are necessary for study inclusion, and thus by methodology, studies included here did report activational changes during the offline scanning point. Indeed the SRTT byGregory et al. (2014)found correlations with next-day improvement, and that ofTung et al. (2013)investigated neural changes as a result of motor-task recency, and thus while the findings may not strictly represent offline motor learning processes, they do implicate regions association with the after-effects or changes to neural activation as a result of motor learning.

### 4.3 Shared activation between online and offline learning

The validation of both online and offline correlates of motor learning here, justify a further conjunction analysis in which we identify clusters which significantly overlap between online and offline regional activation changes. There were two significant clusters (larger than 100 mm^3^) that arose from the analysis, the first being the left postcentral gyrus, and the second being the left supplemental motor area. The implication of the left postcentral gyrus is consistent with motor learning both online and offline, given that it contains the primary somatosensory cortex (DiGuiseppi & Tadi, 2023). Indeed the somatosensory cortex is a contributor to motor memory consolidation, as previous work has shown that theta-burst transcranial magnetic stimulation (cTBS) of the somatosensory cortex interfered with consolidation and eventual retrieval (Kumar et al., 2019). The role of the somatosensory cortex in online motor learning has also been extensively evidenced (Ito et al., 2016;Ostry et al., 2010;Wong et al., 2012), whereby the improvement trajectory of even implicit motor tasks can be altered with somatosensorial disruption (Vidoni et al., 2010). Similarly, the supplementary motor area (SMA) has also been shown to have oscillatory changes reflecting performance improvement at post-training, and repetitive TMS (rTMS) of the SMA, directly after practice, reduced recall during a motor task (Tamaki et al., 2013;Tanaka et al., 2009). During online motor learning, the SMA is generally considered important for motor control (Nachev et al., 2007), and indeed this region was highlighted in the meta-analyses byHardwick et al. (2013)and byDahms et al. (2020). Thus, our findings suggest that while online and offline motor learning correlates have distinct activation which is consistent across numerous task types and populations, there is consistent activation of the SMA and somatosensory cortex across both groups. It should be noted, however, that despite having overlapping regional activation changes, the underlying processes which occur may differ dependent on the motor stage.

### 4.4 Consistency with adaptive online and offline motor learning framework

There is noted concordance between the results presented here, and the proposed adaptive motor learning theory put forth in recent years (Tani et al., 2014). Consistent online activation of the basal ganglia (notably putamen), cerebellum, motor cortices, SMA, and frontal regions can be placed easily within current theories of motor learning activation (Krakauer et al., 2019). This goes beyond the traditional sequence learning, when considering that, despite a majority SRTT, the online motor learning studies included ranged beyond strict sequence learning, and so it is possible that activation of region, consistent with adaptive motor learning theories, is generalizable beyond sequence representation. Similarly, offline activation of regions including basal ganglia and cerebellar nuclei is consistent with error-based learning processes, whereby the cerebellum exerts normal inhibition of the motor cortices via the thalamus (Spampinato & Celnik, 2021). Consistent too, with offline activational patterns representing striatal–cortical network activation, is a noted shift from cerebellar to striatal–cortical network which is thought to represent improved forecasting models in the cerebellum associated with error correction and updating (Dahms et al., 2020). Overall then, our results support signatures consistent with the adaptive motor learning frameworks, and support consistent activation across numerous motor studies with varying tasks, durations, and specifications. We also report numerous instances of activational similarity between online and offline scanning, which suggests that regions associated with adaptive motor processes during learning are similarly engaged after online learning has ceased, but when continued learning processes still occur.

### 4.5 Limitations

Owing to a small number of studies that were eventually included in this analysis, we opted not to perform sub-analyses by the type of task used, as the low number of studies included in each sub-analyses would not provide generalizable results. Thus, our findings should be taken in context of activation which is sufficiently represented across a variety of tasks, and indeed there were clusters which were activated across studies, regardless of task, seeSupplemental Tables.

Regions between bilateral thalami likely did not survive the ALE or conjunction analyses since the individual activations were spread inconsistently across both thalami, and, therefore, producing no clear cluster reaching significance. As a result, the jackknife analysis produced the midpoint of the cluster directly between thalami, suggesting thalamic involvement, but not with notable spatial concentration. Accordingly, the thalamus is also a region of strong consideration for implication in online and offline motor learning, but it is not extensively discussed here.

There is a substantial difference in the literature size sourced for each online and offline group. This is likely due to an (understandable) tendency to assess motor learning correlates concurrent with the task as opposed to independently, which greatly reduces the number of available fMRI studies that investigated consolidation or offline periods. Thus, while here we compare online and offline scanning point findings, there are likely more neural correlates of offline motor learning scanning which did not survive the analysis due to a small sample size, or inconsistent regional activation. Therefore, we highlight a clear imbalance between scanning which investigates online correlates of motor learning, and scanning during offline, post-learning periods.

It must be noted that our task inclusion criteria did not specify task specificity. This choice was intentional so as to approach the question of online/offline motor learning activation in the general sense as opposed to specific to a given effector or conceptual process. In this way, our findings (taken in context with the jackknife analysis) are more representative of general motor learning processes as they relate to online and offline scanning, as opposed to a single motor learning process such as procedural or adaptive learning. While this allows for a more general interpretation about neural activation as it relates to general motor learning, it also prevents more specific predictions about motor learning as it relates to characteristics such as finger vs. arm use, explicit vs implicit knowledge, duration, task, and effort. These individual aspects almost certainly implicate different neural regions, and so individually (if sample size would allow) would produce different effects in a subsequent or future analysis.

As a further limitation, due to sample size, we did not pursue a regression-based analysis which considered the effect of time on the offline motor learning activity. Indeed aspects such as the time of day, sleep, and duration of rest have been shown to influence consolidation processes (Ruffino et al., 2021;Truong et al., 2023), and thus in an analysis with more offline studies, this variable would require consideration to elaborate the effect of offline period following the initial learning.

We, therefore, stress that as a constraint to this work, our findings only narrate the regional implication to online and offline processes in general, highlighting their differences. We also echo the findings by those such asTung et al. (2013), who also stressed the importance of exercising caution when scanning after motor learning tasks, as placement time may prove critical to capture effects which may relate to offline processes or which may represent after-effects of recent motor learning.

## 5 Conclusion

Our findings identify unique and overlapping activation changes associated with online and offline motor learning correlates, supporting previous work and theories about regional contributions to motor learning. To our knowledge, this represents the first instance of a meta-analysis directly comparing online with offline motor learning, highlighting their respective overlapping and distinct signatures. While preliminary and restricted by study numbers, our findings replicate previous meta-analyses which investigated online motor learning, and find support for the theory of the cortico-striatal network’s involvement in motor consolidation and offline processes. By comparing online and offline ALE maps via conjunction analyses, we also concluded that both SMA and somatosensory regions have roles in online and offline motor learning. Overall, we also find evidence for adaptive motor learning processes persisting into offline scanning, likely reflecting consolidatory processes which represented a reactivation of the same adaptative pathways during online learning. Our findings support future work which seeks to investigate overarching processes that govern motor learning, and identifies targets for stimulation and modulation at either online or offline learning stages while supporting current models of motor learning. We also highlight the need for further research into offline motor learning correlates, given the lack of literature in this area reflected by small study availability.

## Supplementary Material

Supplementary Material

## Data Availability

For database search and analysis, NiMARE (https://github.com/neurostuff/NiMARE) was used, integrating both Neurosynth (https://github.com/neurosynth/neurosynth) and Neuroquery (https://github.com/neuroquery) databases. Plotting was performed using the nilearn package (https://github.com/nilearn).

## References

[b1] Albouy , G. , Sterpenich , V. , Balteau , E. , Vandewalle , G. , Desseilles , M. , Dang-Vu , T. , Darsaud , A. , Ruby , P. , Luppi , P. H. , Degueldre , C. , Peigneux , P. , Luxen , A. , & Maquet , P. ( 2008 ). Both the hippocampus and striatum are involved in consolidation of motor sequence memory . Neuron , 58 ( 2 ), 261 – 272 . 10.1016/j.neuron.2008.02.008 18439410

[b2] Andersen , K. W. , & Siebner , H. R. ( 2018 ). Mapping dexterity and handedness: Recent insights and future challenges . Current Opinion in Behavioral Sciences , 20 , 123 – 129 . 10.1016/j.cobeha.2017.12.020

[b3] Anguera , J. A. , Russell , C. A. , Noll , D. C. , & Seidler , R. D. ( 2007 ). Neural correlates associated with intermanual transfer of sensorimotor adaptation . Brain Research , 1185 , 136 – 151 . 10.1016/j.brainres.2007.09.088 17996854

[b4] Arima , T. , Yanagi , Y. , Niddam , D. M. , Ohata , N. , Arendt-Nielsen , L. , Minagi , S. , Sessle , B. J. , & Svensson , P. ( 2011 ). Corticomotor plasticity induced by tongue-task training in humans: A longitudinal fMRI study . Exp Brain Res , 212 ( 2 ), 199 – 212 . 10.1007/s00221-011-2719-7 21590261

[b5] Attwell , P. J. E. , Cooke , S. F. , & Yeo , C. H. ( 2002 ). Cerebellar function in consolidation of a motor memory . Neuron , 34 ( 6 ), 1011 – 1020 . 10.1016/S0896-6273(02)00719-5 12086647

[b6] Bo , J. , Peltier , S. J. , Noll , D. C. , & Seidler , R. D. ( 2011 ). Symbolic representations in motor sequence learning . Neuroimage , 54 ( 1 ), 417 – 426 . 10.1016/j.neuroimage.2010.08.019 20727412 PMC2962690

[b7] Cunningham , D. A. , Machado , A. , Yue , G. H. , Carey , J. R. , & Plow , E. B. ( 2013 ). Functional somatotopy revealed across multiple cortical regions using a model of complex motor task . Brain Res , 1531 , 25 – 36 . 10.1016/j.brainres.2013.07.050 23920009 PMC3931839

[b8] Dahms , C. , Brodoehl , S. , Witte , O. W. , & Klingner , C. M. ( 2020 ). The importance of different learning stages for motor sequence learning after stroke . Hum Brain Mapp , 41 ( 1 ), 270 – 286 . 10.1002/hbm.24793 31520506 PMC7268039

[b9] Debas , K. , Carrier , J. , Barakat , M. , Marrelec , G. , Bellec , P. , Tahar , A. H. , Karni , A. , Ungerleider , L. G. , Benali , H. , & Doyon , J. ( 2014 ). Off-line consolidation of motor sequence learning results in greater integration within a cortico-striatal functional network . Neuroimage , 99 , 50 – 58 . 10.1016/j.neuroimage.2014.05.022 24844748 PMC6644011

[b10] Debas , K. , Carrier , J. , Orban , P. , Barakat , M. , Lungu , O. , Vandewalle , G. , Tahar , A. H. , Bellec , P. , Karni , A. , & Ungerleider , L. G. ( 2010 ). Brain plasticity related to the consolidation of motor sequence learning and motor adaptation . Proc Natil Acad Sci , 107 ( 41 ), 17839 – 17844 . 10.1073/pnas.1013176107 PMC2955095 20876115

[b11] Di Rienzo , F. , Debarnot , U. , Daligault , S. , Saruco , E. , Delpuech , C. , Doyon , J. , Collet , C. , & Guillot , A. ( 2016 ). Online and offline performance gains following motor imagery practice: A comprehensive review of behavioral and neuroimaging studies . Front Human Neurosci , 10 , 315 . 10.3389/fnhum.2016.00315 PMC4923126 27445755

[b12] DiGuiseppi , J. , & Tadi , P. ( 2023 ). Neuroanatomy, postcentral gyrus . In StatPearls . StatPearls Publishing Copyright © 2023, StatPearls Publishing LLC . Bookshelf ID: NBK549825; PMID: 31751015 . 31751015

[b13] Dockès , J. , Poldrack , R. A. , Primet , R. , Gözükan , H. , Yarkoni , T. , Suchanek , F. , Thirion , B. , & Varoquaux , G. ( 2020 ). NeuroQuery, comprehensive meta-analysis of human brain mapping . eLife , 9 , e53385 . 10.7554/eLife.53385 32129761 PMC7164961

[b14] Doyon , J. , & Benali , H. ( 2005 ). Reorganization and plasticity in the adult brain during learning of motor skills . Curr Opin Neurobiol , 15 ( 2 ), 161 – 167 . 10.1016/j.conb.2005.03.004 15831397

[b15] Drobyshevsky , A. , Baumann , S. B. , & Schneider , W. ( 2006 ). A rapid fMRI task battery for mapping of visual, motor, cognitive, and emotional function . NeuroImage , 31 ( 2 ), 732 – 744 . 10.1016/j.neuroimage.2005.12.016 16488627 PMC1620013

[b16] Ebrahimi , S. , & Ostry , D. J. ( 2024 ). The human somatosensory cortex contributes to the encoding of newly learned movements . Proc Natl Acad Sci , 121 ( 6 ), e2316294121 . 10.1073/pnas.2316294121 38285945 PMC10861869

[b17] Ehsani , F. , Bakhtiary , A. H. , Jaberzadeh , S. , Talimkhani , A. , & Hajihasani , A. ( 2016 ). Differential effects of primary motor cortex and cerebellar transcranial direct current stimulation on motor learning in healthy individuals: A randomized double-blind sham-controlled study . Neurosci Res , 112 , 10 – 19 . 10.1016/j.neures.2016.06.003 27349154

[b18] Eickhoff , S. B. , Laird , A. R. , Grefkes , C. , Wang , L. E. , Zilles , K. , & Fox , P. T. ( 2009 ). Coordinate-based activation likelihood estimation meta-analysis of neuroimaging data: A random-effects approach based on empirical estimates of spatial uncertainty . Hum Brain Mapp , 30 ( 9 ), 2907 – 2926 . 10.1002/hbm.20718 19172646 PMC2872071

[b19] Fernández-Seara , M. A. , Aznárez-Sanado , M. , Mengual , E. , Loayza , F. R. , & Pastor , M. A. ( 2009 ). Continuous performance of a novel motor sequence leads to highly correlated striatal and hippocampal perfusion increases . Neuroimage , 47 ( 4 ), 1797 – 1808 . 10.1016/j.neuroimage.2009.05.061 19481611

[b20] Gheysen , F. , Van Opstal , F. , Roggeman , C. , Van Waelvelde , H. , & Fias , W. ( 2010 ). Hippocampal contribution to early and later stages of implicit motor sequence learning . Exp Brain Res , 202 , 795 – 807 . 10.1007/s00221-010-2186-6 20195849

[b21] Ghilardi , M.-F. , Ghez , C. , Dhawan , V. , Moeller , J. , Mentis , M. , Nakamura , T. , Antonini , A. , & Eidelberg , D. ( 2000 ). Patterns of regional brain activation associated with different forms of motor learning . Brain Res , 871 ( 1 ), 127 – 145 . 10.1016/S0006-8993(00)02365-9 10882792

[b22] Grafton , S. T. , Schmitt , P. , Van Horn , J. , & Diedrichsen , J. ( 2008 ). Neural substrates of visuomotor learning based on improved feedback control and prediction . Neuroimage , 39 ( 3 ), 1383 – 1395 . 10.1016/j.neuroimage.2007.09.062 18032069 PMC2268716

[b23] Gregory , M. D. , Agam , Y. , Selvadurai , C. , Nagy , A. , Vangel , M. , Tucker , M. , Robertson , E. M. , Stickgold , R. , & Manoach , D. S. ( 2014 ). Resting state connectivity immediately following learning correlates with subsequent sleep-dependent enhancement of motor task performance . Neuroimage , 102 , 666 – 673 . 10.1016/j.neuroimage.2014.08.044 25173415 PMC4252600

[b24] Grosbras , M. H. , Beaton , S. , & Eickhoff , S. B. ( 2012 ). Brain regions involved in human movement perception: A quantitative voxel‐based meta‐analysis . Hum Brain Mapp , 33 ( 2 ), 431 – 454 . 10.1002/hbm.21222 21391275 PMC6869986

[b25] Gryga , M. , Taubert , M. , Dukart , J. , Vollmann , H. , Conde , V. , Sehm , B. , Villringer , A. , & Ragert , P. ( 2012 ). Bidirectional gray matter changes after complex motor skill learning . Front Syst Neurosci , 6 , 37 . 10.3389/fnsys.2012.00037 22623914 PMC3353266

[b26] Gupta , M. W. , & Rickard , T. C. ( 2024 ). Comparison of online, offline, and hybrid hypotheses of motor sequence learning using a quantitative model that incorporate reactive inhibition . Sci Rep , 14 ( 1 ), 4661 . 10.1038/s41598-024-52726-9 38409296 PMC11269601

[b27] Hardwick , R. M. , Rottschy , C. , Miall , R. C. , & Eickhoff , S. B. ( 2013 ). A quantitative meta-analysis and review of motor learning in the human brain . Neuroimage , 67 , 283 – 297 . 10.1016/j.neuroimage.2012.11.020 23194819 PMC3555187

[b28] Heitger , M. H. , Ronsse , R. , Dhollander , T. , Dupont , P. , Caeyenberghs , K. , & Swinnen , S. P. ( 2012 ). Motor learning-induced changes in functional brain connectivity as revealed by means of graph-theoretical network analysis . Neuroimage , 61 ( 3 ), 633 – 650 . 10.1016/j.neuroimage.2012.03.067 22503778

[b29] Hétu , S. , Grégoire , M. , Saimpont , A. , Coll , M.-P. , Eugène , F. , Michon , P.-E. , & Jackson , P. L. ( 2013 ). The neural network of motor imagery: An ALE meta-analysis . Neurosci Biobehav Rev , 37 ( 5 ), 930 – 949 . 10.1016/j.neubiorev.2013.03.017 23583615

[b30] Heun , R. , Freymann , N. , Granath , D. O. , Stracke , C. P. , Jessen , F. , Barkow , K. , & Reul , J. ( 2004 ). Differences of cerebral activation between superior and inferior learners during motor sequence encoding and retrieval . Psychiatry Res , 132 ( 1 ), 19 – 32 . 10.1016/j.pscychresns.2004.01.007 15546700

[b31] Ito , T. , Coppola , J. H. , & Ostry , D. J. ( 2016 ). Speech motor learning changes the neural response to both auditory and somatosensory signals . Sci Rep , 6 ( 1 ), 25926 . 10.1038/srep25926 27181603 PMC4867601

[b32] Karim , H. T. , Huppert , T. J. , Erickson , K. I. , Wollam , M. E. , Sparto , P. J. , Sejdić , E. , & VanSwearingen , J. M. ( 2017 ). Motor sequence learning-induced neural efficiency in functional brain connectivity . Behav Brain Res , 319 , 87 – 95 . 10.1016/j.bbr.2016.11.021 27845228 PMC5183470

[b33] Kornysheva , K. , & Diedrichsen , J. ( 2014 ). Human premotor areas parse sequences into their spatial and temporal features . eLife , 3 , e03043 . 10.7554/eLife.03043 25117541 PMC4123716

[b34] Krakauer , J. W. , Hadjiosif , A. M. , Xu , J. , Wong , A. L. , & Haith , A. M. ( 2019 ). Motor learning . Compr Physiol , 9 ( 2 ), 613 – 663 . 10.1002/cphy.c170043 30873583

[b35] Krakauer , J. W. , & Shadmehr , R. ( 2006 ). Consolidation of motor memory . Trends Neurosci , 29 ( 1 ), 58 – 64 . 10.1016/j.tins.2005.10.003 16290273 PMC2553888

[b36] Kumar , N. , Manning , T. F. , & Ostry , D. J. ( 2019 ). Somatosensory cortex participates in the consolidation of human motor memory . PLoS Biol , 17 ( 10 ), e3000469 . 10.1371/journal.pbio.3000469 31613874 PMC6793938

[b37] Lacroix , A. , Proulx-Bégin , L. , Hamel , R. , De Beaumont , L. , Bernier , P.-M. , & Lepage , J.-F. ( 2019 ). Static magnetic stimulation of the primary motor cortex impairs online but not offline motor sequence learning . Sci Rep , 9 ( 1 ), 9886 . 10.1038/s41598-019-46379-2 31285526 PMC6614538

[b38] Lefebvre , S. , Dricot , L. , Gradkowski , W. , Laloux , P. , & Vandermeeren , Y. ( 2012 ). Brain activations underlying different patterns of performance improvement during early motor skill learning . Neuroimage , 62 ( 1 ), 290 – 299 . 10.1016/j.neuroimage.2012.04.052 22569545

[b39] Lissek , S. , Vallana , G. S. , Güntürkün , O. , Dinse , H. , & Tegenthoff , M. ( 2013 ). Brain activation in motor sequence learning is related to the level of native cortical excitability . PLoS One , 8 ( 4 ), e61863 . 10.1371/journal.pone.0061863 23613956 PMC3628854

[b40] Luft , A. R. , & Buitrago , M. M. ( 2005 ). Stages of motor skill learning . Mol Neurobiol , 32 ( 3 ), 205 – 216 . 10.1385/MN:32:3:205 16385137

[b41] Ma , L. , Narayana , S. , Robin , D. A. , Fox , P. T. , & Xiong , J. ( 2011 ). Changes occur in resting state network of motor system during 4 weeks of motor skill learning . Neuroimage , 58 ( 1 ), 226 – 233 . 10.1016/j.neuroimage.2011.06.014 21689765 PMC3144281

[b42] Macintosh , B. J. , Mraz , R. , McIlroy , W. E. , & Graham , S. J. ( 2007 ). Brain activity during a motor learning task: An fMRI and skin conductance study . Hum Brain Mapp , 28 ( 12 ), 1359 – 1367 . 10.1002/hbm.20351 17318835 PMC4896816

[b43] Magdoom , K. N. , Subramanian , D. , Chakravarthy , V. S. , Ravindran , B. , Amari , S.-i. , & Meenakshisundaram , N. ( 2011 ). Modeling basal ganglia for understanding Parkinsonian reaching movements . Neural Comput , 23 ( 2 ), 477 – 516 . 10.1162/NECO_a_00073 21105828

[b44] Marinelli , L. , Quartarone , A. , Hallett , M. , Frazzitta , G. , & Ghilardi , M. F. ( 2017 ). The many facets of motor learning and their relevance for Parkinson’s disease . Clin Neurophysiol , 128 ( 7 ), 1127 – 1141 . 10.1016/j.clinph.2017.03.042 28511125 PMC5486221

[b45] Mary , A. , Wens , V. , Op de Beeck , M. , Leproult , R. , De Tiège , X. , & Peigneux , P. ( 2017 ). Resting-state functional connectivity is an age-dependent predictor of motor learning abilities . Cereb Cortex , 27 ( 10 ), 4923 – 4932 . 10.1093/cercor/bhw286 27655931

[b46] Melcher , T. , Winter , D. , Hommel , B. , Pfister , R. , Dechent , P. , & Gruber , O. ( 2013 ). The neural substrate of the ideomotor principle revisited: Evidence for asymmetries in action-effect learning . Neuroscience , 231 , 13 – 27 . 10.1016/j.neuroscience.2012.11.035 23206874

[b47] Muellbacher , W. , Ziemann , U. , Wissel , J. , Dang , N. , Kofler , M. , Facchini , S. , Boroojerdi , B. , Poewe , W. , & Hallett , M. ( 2002 ). Early consolidation in human primary motor cortex . Nature , 415 ( 6872 ), 640 – 644 . 10.1038/nature712 11807497

[b48] Müller , R.-A. , Kleinhans , N. , Pierce , K. , Kemmotsu , N. , & Courchesne , E. ( 2002 ). Functional MRI of motor sequence acquisition: Effects of learning stage and performance . Cogn Brain Res , 14 ( 2 ), 277 – 293 . 10.1016/S0926-6410(02)00131-3 12067701

[b49] Nachev , P. , Wydell , H. , O’Neill , K. , Husain , M. , & Kennard , C. ( 2007 ). The role of the pre-supplementary motor area in the control of action . Neuroimage , 36 , T155 – T163 . 10.1016/j.neuroimage.2007.03.034 17499162 PMC2648723

[b50] Oishi , K. , Toma , K. , Bagarinao , E. T. , Matsuo , K. , Nakai , T. , Chihara , K. , & Fukuyama , H. ( 2005 ). Activation of the precuneus is related to reduced reaction time in serial reaction time tasks . Neurosci Res , 52 ( 1 ), 37 – 45 . 10.1016/j.neures.2005.01.008 15811551

[b51] Olson , I. R. , Rao , H. , Moore , K. S. , Wang , J. , Detre , J. A. , & Aguirre , G. K. ( 2006 ). Using perfusion fMRI to measure continuous changes in neural activity with learning . Brain Cogn , 60 ( 3 ), 262 – 271 . 10.1016/j.bandc.2005.11.010 16423439

[b52] Orban , P. , Peigneux , P. , Lungu , O. , Albouy , G. , Breton , E. , Laberenne , F. , Benali , H. , Maquet , P. , & Doyon , J. ( 2010 ). The multifaceted nature of the relationship between performance and brain activity in motor sequence learning . Neuroimage , 49 ( 1 ), 694 – 702 . 10.1016/j.neuroimage.2009.08.055 19732838

[b53] Orban , P. , Peigneux , P. , Lungu , O. , Debas , K. , Barakat , M. , Bellec , P. , Benali , H. , Maquet , P. , & Doyon , J. ( 2011 ). Functional neuroanatomy associated with the expression of distinct movement kinematics in motor sequence learning . Neuroscience , 179 , 94 – 103 . 10.1016/j.neuroscience.2011.01.040 21277942

[b54] Ostry , D. J. , Darainy , M. , Mattar , A. A. , Wong , J. , & Gribble , P. L. ( 2010 ). Somatosensory plasticity and motor learning . J Neurosci , 30 ( 15 ), 5384 – 5393 . 10.1523/jneurosci.4571-09.2010 20392960 PMC2858322

[b55] Pammi , V. S. , Miyapuram , K. P. , Ahmed , Samejima, K. , Bapi , R. S. , & Doya , K. ( 2012 ). Changing the structure of complex visuo-motor sequences selectively activates the fronto-parietal network . Neuroimage , 59 ( 2 ), 1180 – 1189 . 10.1016/j.neuroimage.2011.08.006 21867758

[b56] Papale , A. E. , & Hooks , B. M. ( 2018 ). Circuit changes in motor cortex during motor skill learning . Neuroscience , 368 , 283 – 297 . 10.1016/j.neuroscience.2017.09.010 28918262 PMC5762136

[b57] Penhune , V. B. , & Doyon , J. ( 2005 ). Cerebellum and M1 interaction during early learning of timed motor sequences . Neuroimage , 26 ( 3 ), 801 – 812 . 10.1016/j.neuroimage.2005.02.041 15955490

[b58] Pollok , B. , Keitel , A. , Foerster , M. , Moshiri , G. , Otto , K. , & Krause , V. ( 2020 ). The posterior parietal cortex mediates early offline-rather than online-motor sequence learning . Neuropsychologia , 146 , 107555 . 10.1016/j.neuropsychologia.2020.107555 32653440

[b59] Porrill , J. , & Dean , P. ( 2007 ). Recurrent cerebellar loops simplify adaptive control of redundant and nonlinear motor systems . Neural Comput , 19 ( 1 ), 170 – 193 . 10.1162/neco.2007.19.1.170 17134321

[b60] Rémy , F. , Wenderoth , N. , Lipkens , K. , & Swinnen , S. P. ( 2008 ). Acquisition of a new bimanual coordination pattern modulates the cerebral activations elicited by an intrinsic pattern: An fMRI study . Cortex , 44 ( 5 ), 482 – 493 . 10.1016/j.cortex.2007.07.004 18387582

[b61] Roshchupkina , L. , Wens , V. , Coquelet , N. , de Tiege , X. , & Peigneux , P. ( 2022 ). Resting state fast brain dynamics predict interindividual variability in motor performance . Sci Rep , 12 ( 1 ), 5340 . 10.1038/s41598-022-08767-z 35351907 PMC8964712

[b62] Ruffino , C. , Truong , C. , Dupont , W. , Bouguila , F. , Michel , C. , Lebon , F. , & Papaxanthis , C. ( 2021 ). Acquisition and consolidation processes following motor imagery practice . Sci Rep , 11 ( 1 ), 2295 . 10.1038/s41598-021-81994-y 33504870 PMC7840673

[b63] Sacco , K. , Cauda , F. , D’Agata , F. , Mate , D. , Duca , S. , & Geminiani , G. ( 2009 ). Reorganization and enhanced functional connectivity of motor areas in repetitive ankle movements after training in locomotor attention . Brain Res , 1297 , 124 – 134 . 10.1016/j.brainres.2009.08.049 19703428

[b64] Salo , T. , Yarkoni , T. , Nichols , T. E. , Poline , J.-B. , Kent , J. D. , Gorgolewski , K. J. , Glerean , E. , Bottenhorn , K. L. , Bilgel , M. , Wright , J. , Reeders , P. , Kimbler , A. , Nielson , D. N. , Yanes , J. A. , Pérez , A. , Oudyk , K. M. , Jarecka , D. , Enge , A. , Peraza , J. A. , … Laird , A. R . ( 2023 ). neurostuff/NiMARE: 0.0.13 . In Zenodo. 10.55458/neurolibre.00007

[b65] Sami , S. , & Miall , R. ( 2013 ). Graph network analysis of immediate motor-learning induced changes in resting state BOLD . Front Human Neurosci , 7 , 166 . 10.3389/fnhum.2013.00166 PMC3654214 23720616

[b66] Schlund , M. W. , Rosales-Ruiz , J. , Vaidya , M. , Glenn , S. S. , & Staff , D. ( 2008 ). Experience-dependent plasticity: Differential changes in activation associated with repeated reinforcement . Neuroscience , 155 ( 1 ), 17 – 23 . 10.1016/j.neuroscience.2008.04.076 18565682

[b67] Seidler , R. D. , Bo , J. , & Anguera , J. A. ( 2012 ). Neurocognitive contributions to motor skill learning: The role of working memory . J Mot Behav , 44 ( 6 ), 445 – 453 . 10.1080/00222895.2012.672348 23237467 PMC3534841

[b68] Shadmehr , R. , & Brashers-Krug , T. ( 1997 ). Functional stages in the formation of human long-term motor memory . J Neurosci , 17 ( 1 ), 409 – 419 . 10.1523/jneurosci.17-01-00409.1997 8987766 PMC6793707

[b69] Shadmehr , R. , & Krakauer , J. W. ( 2008 ). A computational neuroanatomy for motor control . Exp Brain Res , 185 ( 3 ), 359 – 381 . 10.1007/s00221-008-1280-5 18251019 PMC2553854

[b70] Shadmehr , R. , Smith , M. A. , & Krakauer , J. W. ( 2010 ). Error correction, sensory prediction, and adaptation in motor control . Annu Rev Neurosci , 33 ( 1 ), 89 – 108 . 10.1146/annurev-neuro-060909-153135 20367317

[b71] Sidarta , A. , Vahdat , S. , Bernardi , N. F. , & Ostry , D. J. ( 2016 ). Somatic and reinforcement-based plasticity in the initial stages of human motor learning . J Neurosci , 36 ( 46 ), 11682 – 11692 . 10.1523/jneurosci.1767-16.2016 27852776 PMC5125226

[b72] Spampinato , D. , & Celnik , P. ( 2021 ). Multiple motor learning processes in humans: Defining their neurophysiological bases . Neuroscientist , 27 ( 3 ), 246 – 267 . 10.1177/1073858420939552 32713291 PMC8151555

[b73] Tamaki , M. , Huang , T.-R. , Yotsumoto , Y. , Hämäläinen , M. , Lin , F.-H. , José E. Náñez , S. , Watanabe , T. , & Sasaki , Y. ( 2013 ). Enhanced spontaneous oscillations in the supplementary motor area are associated with sleep-dependent offline learning of finger-tapping motor-sequence task . J Neurosci , 33 ( 34 ), 13894 – 13902 . 10.1523/jneurosci.1198-13.2013 23966709 PMC3755724

[b74] Tamás Kincses , Z. , Johansen-Berg , H. , Tomassini , V. , Bosnell , R. , Matthews , P. M. , & Beckmann , C. F. ( 2008 ). Model-free characterization of brain functional networks for motor sequence learning using fMRI . Neuroimage , 39 ( 4 ), 1950 – 1958 . 10.1016/j.neuroimage.2007.09.070 18053746

[b75] Tanaka , S. , Honda , M. , Hanakawa , T. , & Cohen , L. G. ( 2009 ). Differential contribution of the supplementary motor area to stabilization of a procedural motor skill acquired through different practice schedules . Cereb Cortex , 20 ( 9 ), 2114 – 2121 . 10.1093/cercor/bhp276 20038545 PMC2923213

[b76] Tani , G. , Corrêa , U. C. , Basso , L. , Benda , R. N. , Ugrinowitsch , H. , & Choshi , K. ( 2014 ). An adaptive process model of motor learning: Insights for the teaching of motor skills . Nonlinear Dynamics Psychol Life Sci , 18 ( 1 ), 47 – 65 . 10.5628/rpcd.10.01.158 24314130

[b77] Tomassini , V. , Jbabdi , S. , Kincses , Z. T. , Bosnell , R. , Douaud , G. , Pozzilli , C. , Matthews , P. M. , & Johansen‐Berg , H. ( 2011 ). Structural and functional bases for individual differences in motor learning . Hum Brain Mapp , 32 ( 3 ), 494 – 508 . 10.1002/hbm.21037 20533562 PMC3674543

[b78] Tracy , J. I. , Faro , S. S. , Mohammed , F. , Pinus , A. , Christensen , H. , & Burkland , D. ( 2001 ). A comparison of ‘Early’ and ‘Late’ stage brain activation during brief practice of a simple motor task . Brain Res Cogn Brain Res , 10 ( 3 ), 303 – 316 . 10.1016/s0926-6410(00)00045-8 11167053

[b79] Truong , C. , Ruffino , C. , Gaveau , J. , White , O. , Hilt , P. M. , & Papaxanthis , C. ( 2023 ). Time of day and sleep effects on motor acquisition and consolidation . NPJ Sci Learn , 8 ( 1 ), 30 . 10.1038/s41539-023-00176-9 37658041 PMC10474136

[b80] Tung , K. C. , Uh , J. , Mao , D. , Xu , F. , Xiao , G. , & Lu , H. ( 2013 ). Alterations in resting functional connectivity due to recent motor task . Neuroimage , 78 , 316 – 324 . 10.1016/j.neuroimage.2013.04.006 23583747 PMC3672369

[b81] Tunovic , S. , Press , D. Z. , & Robertson , E. M. ( 2014 ). A physiological signal that prevents motor skill improvements during consolidation . J Neurosci , 34 ( 15 ), 5302 – 5310 . 10.1523/jneurosci.3497-13.2014 24719108 PMC3983806

[b82] Tzourio-Mazoyer , N. , Landeau , B. , Papathanassiou , D. , Crivello , F. , Etard , O. , Delcroix , N. , Mazoyer , B. , & Joliot , M. ( 2002 ). Automated anatomical labeling of activations in SPM using a macroscopic anatomical parcellation of the MNI MRI single-subject brain . NeuroImage , 15 ( 1 ), 273 – 289 . 10.1006/nimg.2001.0978 11771995

[b83] Tzvi , E. , Münte , T. F. , & Krämer , U. M. ( 2014 ). Delineating the cortico-striatal-cerebellar network in implicit motor sequence learning . Neuroimage , 94 , 222 – 230 . 10.1016/j.neuroimage.2014.03.004 24632466

[b84] Vahdat , S. , Darainy , M. , Milner , T. E. , & Ostry , D. J. ( 2011 ). Functionally specific changes in resting-state sensorimotor networks after motor learning . J Neurosci , 31 ( 47 ), 16907 – 16915 . 10.1523/jneurosci.2737-11.2011 22114261 PMC3260885

[b85] Verhoeven , F. M. , & Newell , K. M. ( 2018 ). Unifying practice schedules in the timescales of motor learning and performance . Hum Mov Sci , 59 , 153 – 169 . 10.1016/j.humov.2018.04.004 29684760

[b86] Vidoni , E. D. , Acerra , N. E. , Dao , E. , Meehan , S. K. , & Boyd , L. A. ( 2010 ). Role of the primary somatosensory cortex in motor learning: An rTMS study . Neurobiol Learn Mem , 93 ( 4 ), 532 – 539 . 10.1016/j.nlm.2010.01.011 20132902

[b87] Wali , M. ( 2020 ). Role of the somatosensory cortex in motor memory consolidation . J Neurophysiol , 124 ( 3 ), 648 – 651 . 10.1152/jn.00770.2019 32727313

[b88] Watanabe , R. , Watanabe , S. , Kuruma , H. , Murakami , Y. , Seno , A. , & Matsuda , T. ( 2011 ). Neural activation during imitation of movements presented from four different perspectives: A functional magnetic resonance imaging study . Neurosci Lett , 503 ( 2 ), 100 – 104 . 10.1016/j.neulet.2011.08.016 21871533

[b89] Wong , J. D. , Kistemaker , D. A. , Chin , A. , & Gribble , P. L. ( 2012 ). Can proprioceptive training improve motor learning? J Neurophysiol , 108 ( 12 ), 3313 – 3321 . 10.1152/jn.00122.2012 22972960 PMC3544879

[b90] Yarkoni , T. , Poldrack , R. A. , Nichols , T. E. , Van Essen , D. C. , & Wager , T. D. ( 2011 ). Large-scale automated synthesis of human functional neuroimaging data . Nat Methods , 8 ( 8 ), 665 – 670 . 10.1038/nmeth.1635 21706013 PMC3146590

[b91] Zapparoli , L. , Mariano , M. , & Paulesu , E. ( 2022 ). How the motor system copes with aging: A quantitative meta-analysis of the effect of aging on motor function control . Commun Biol , 5 ( 1 ), 79 . 10.1038/s42003-022-03027-2 35058549 PMC8776875

